# A Content Framework of a Novel Patient-Reported Outcome Measure for Detecting Early Adverse Events After Major Abdominal Surgery

**DOI:** 10.1007/s00268-023-07143-w

**Published:** 2023-08-23

**Authors:** Johan Clausen, Victor Boesen, Ismail Gögenur, Torquil Watt

**Affiliations:** 1 Center for Surgical Science, Surgical Department, Zealand’s University Hospital, Lykkebaekvej 1, 4600 Koege, Denmark; 2 Department of Endocrinology, Gentofte and Herlev Hospital, Borgmester Ib Juuls Vej 1, 2730 Herlev, Denmark

## Abstract

**Background:**

Systematic health monitoring with patient-reported outcome instruments may predict post-discharge complications after major surgery. The objective of this study was to conceptualize a novel patient-reported outcome instrument for detecting early adverse events within two weeks of discharge after major emergency abdominal surgery and colorectal cancer surgery.

**Methods:**

This study was conducted in two phases. (1) An exhaustive health concept pool was generated using systematic content analysis of existing patient-reported outcome measures (*N* = 31) and semi-structured interviews of readmitted patients (*N* = 49) and health professionals (*N* = 10). Concepts were categorized into three major domains: ‘Symptoms,’ ‘functional status,’ and ‘general health perception.’ We calculated the frequency of each health concept as the proportion of patients, who experienced the respective concept prior to readmission. (2) Colorectal cancer surgeons (*N *= 13) and emergency general surgeons (*N* = 12) rated the relevance of each health concept (1 = irrelevant, 5 = very relevant) in the context of detecting post-discharge adverse events. We selected concepts with either a high mean relevance score (≥ 4) or a combination of moderate mean relevance score and high patient-reported frequency (≥ 3 and ≥ 20% or ≥ 2.5 and ≥ 50%, respectively).

**Results:**

Content analysis of existing items with additions from patients and experts resulted in 58 health concepts, of which the majority were distinct symptoms (*N* = 40). The selection procedure resulted in 29 patient-reported health concepts relevant for detecting adverse events after discharge.

**Conclusion:**

The outlined framework provides content validity for future patient-reported outcome instruments detecting adverse events in the early post-discharge period after major emergency abdominal surgery and colorectal cancer surgery.

**Supplementary Information:**

The online version contains supplementary material available at 10.1007/s00268-023-07143-w.

## Introduction

Major abdominal surgery carries a significant risk of postoperative complications, which have an adverse impact on long-term prognosis and hospital expenses [1, 2]. The introduction of enhanced recovery after surgery (ERAS) programs has markedly improved recovery and shortened length of stay; however, initiatives to improve post-discharge quality of care after abdominal surgery remain sparse [3]. Unplanned 30-day readmission is reported to occur in 9–25% of patients undergoing general abdominal and colorectal surgery and is strongly correlated with post-discharge adverse events [4–7]. Systematic symptom monitoring after discharge might improve self-managing support and assist healthcare professionals in tailoring appropriate out-patient interventions, ultimately reducing morbidity and hospital expenses [8–10]. However, patient-reported outcome measures (PROM) specifically developed for detecting postoperative complications in the early post-discharge period are lacking.

The extent to which a PROM measures all important aspects of the outcome of interest (i.e., content validity) is a crucial part of PROM validity and should be established early in the development phase and prior to quantitative psychometric testing [11, 12]. Current guidelines on PROM development recommend that inputs from existing literature, experts in the field, and patients from the target population should be conceptualized in a framework that provides a structural outline of the PROM content [11–14].

The objective of this study was to develop a comprehensive content framework and thereby ensuring content validity of a future electronic PROM (ePROM) intended for early detection of adverse events occurring within 14 days after discharge from colorectal cancer (CRC) surgery or high-risk emergency open or laparoscopic surgery involving the stomach, small intestines, colon, or rectum.

## Material and methods

### Design

**Fig. 1:**
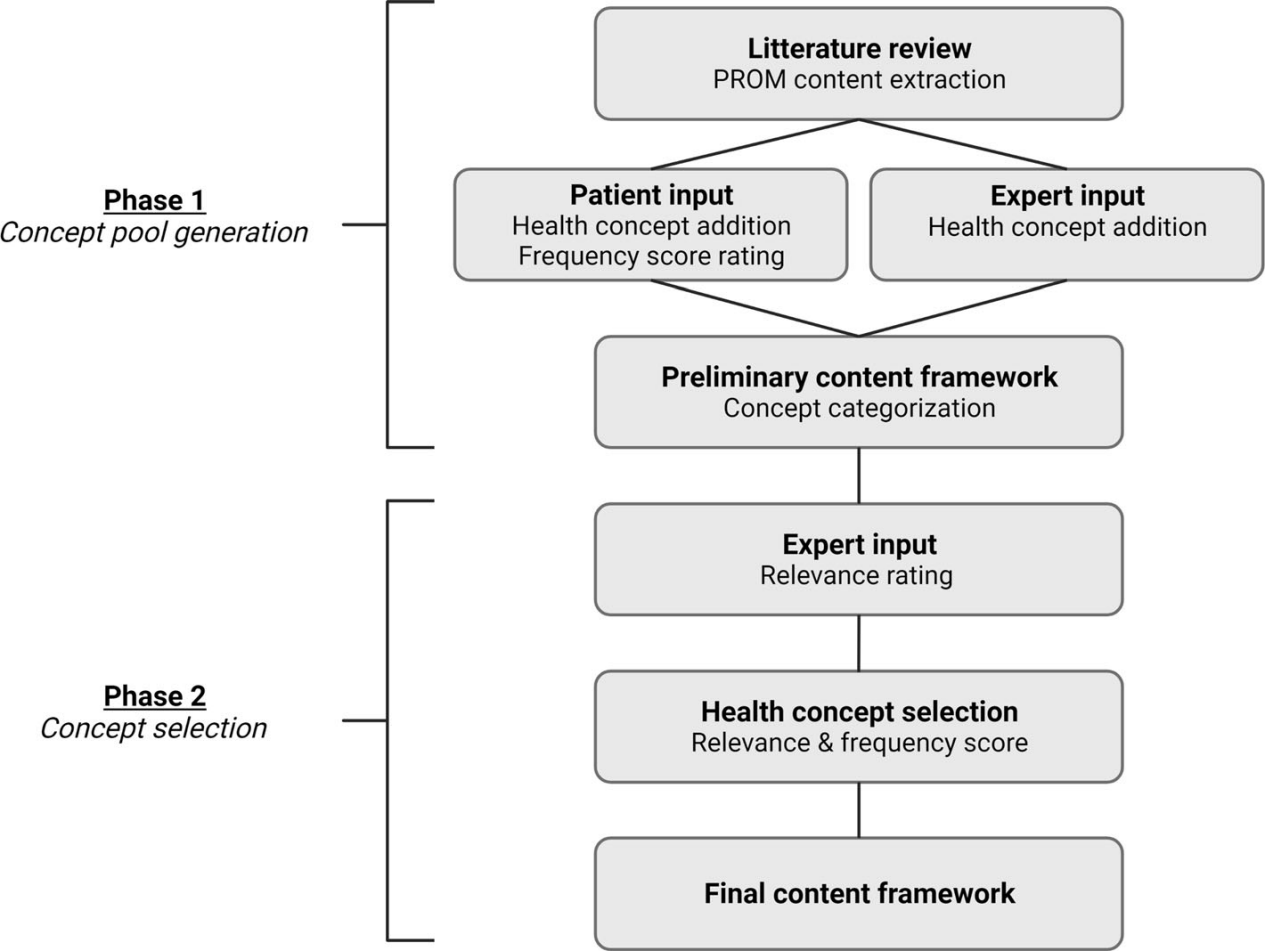
Study flowchart illustrating the two phases of the study. PROM: Patient-reported outcome measure

The study was designed as a mixed method study (Fig. 1). An initial concept pool generation phase (phase 1) was conducted followed by a concept selection phase (phase 2), which are described in detail below.

### Phase 1: concept pool generation

The purpose of this phase was to generate an exhaustive pool of patient-reported health concepts with possible relevance for detecting postoperative adverse events. As recommended in existing guidelines on PROM development [13, 14], we integrated inputs from existing literature, patients, and experts in the field.

### Literature review

Existing PROMs measuring multidimensional recovery after abdominal surgery were identified using a recent systematic review [15]. In addition, we further identified PROMs measuring domain, organ, disease, or procedure-specific recovery or health-related quality of life (HRQOL) after abdominal surgery by examining the review’s online appendix and reference list supplemented by a literature search using pubmed.gov and the Patient-Reported Outcome and Quality of life Instruments Database. We only included PROMs developed or psychometrically tested in the context of abdominal surgery. PROMs were excluded if we were not able to retrieve the original instrument or if items were not described in detail in key publications. Items from all included PROMs were extracted verbatim and pooled. A clinically founded content analysis was performed to isolate the underlying health concept measured by each item. Item content was initially mapped to WHO’s international classification of diseases (ICD-10) or WHO’s International Classification of Functioning, Disability, and Health (ICF) index [16]. Custom concept categories were created for items without a suitable ICD-10 or ICF category.

### Hypothesized conceptual framework

Identified health concepts were categorized into ‘symptoms,’ ‘functional status,’ or ‘general health perception’ using a clinical, content-based approach. The definition of the major health domains was based on the widely used taxonomy of health outcomes originally outlined by Wilson and Cleary [17]. As proposed in the original outline, we hypothesized a downstream causal relationship between the three health domains. In a subsequent qualitative refinement process, concepts were translated into Danish. Closely related concepts were merged. Concepts assessed as unfeasible for self-reporting (e.g., somnolence) or requiring biological measurements (e.g., weight) were excluded.

### Patient recruitment

We retrospectively identified all patients, who underwent acute readmission within 30 days after discharge from the surgical department of Zealand University Hospital in Denmark. Patients undergoing either CRC surgery or major emergency abdominal surgery between 01-02-2020 and 01-02-2021 were included. CRC surgery was defined using a combination of ICD-10 CRC codes and the Danish version of the Nordic Medico-Statistical Committee classification of Surgical Procedures (NCSP) codes (online resource 1). Patients undergoing elective and emergency CRC surgery were eligible for inclusion. Major emergency abdominal surgery was defined as in the bundle-care protocol ‘Optimizing major emergency abdominal surgery (OMEGA),’ which was established in 2017 as a local quality-assurance initiative to reduce treatment delays and perioperative morbidity. The protocol consisted of standardized perioperative treatment initiatives for all adult patients (≥ 18 years), who underwent a high-risk emergency surgical procedure involving the stomach, small intestine, colon, or rectum within 72 h of hospital admission or as an in-hospital surgical re-intervention. Patients, who underwent open or laparoscopic procedures for acute conditions such as bowel obstruction, ischemia, intraabdominal bleeding, hollow organ perforation, intraabdominal abscess, or fascial dehiscence were included in the OMEGA protocol. The OMEGA initiatives are described in detail elsewhere [18].

Study exclusion criteria were endoscopy as the definitive procedure, pregnancy, language barriers, cognitive dysfunction, non-acute readmission, or death before the time of electronic health record (EHR) data extraction.

### Patient and expert interviews

The study investigator (JC) contacted all eligible patients by telephone between 16-02-2021 and 02-03-2021 and asked for participation in the study. A semi-structured, individual phone interview with open questions was conducted to explore the patients’ perspectives on experienced symptoms, emotions, functional status, and overall health condition in the days before the unplanned readmission (online resource 2). Subsequently, the patients were asked to review the categorized list of health concepts and add any concept they might find relevant. In addition, the patients were asked to note if they experienced any of the listed health concepts prior to their readmission (yes or no). For each concept, a patient frequency score was calculated as the proportion of patients who stated that they experienced a deterioration in the respective concept.

An expert panel of three colorectal surgeons, two emergency general surgeons, three surgical nurses, and two nurses specialized in treating stoma-related issues reviewed the categorized concept list. The experts were asked to add patient-reported health concepts that might deteriorate prior to postoperative adverse events in a post-discharge setting.

### Phase 2: health concept selection

We invited CRC surgeons and emergency general surgeons from four Danish hospitals to rate all identified health concepts by relevance. Participating surgeons were asked to include both the concept frequency (i.e., the expected incidence of the health concept in the target population) and concept importance (i.e., the extent to which the concept was expected to deteriorate prior to a postoperative complication) in their rating of concept relevance. Concepts were rated on a 5-point Likert scale (1 = irrelevant, 5 = very relevant). The surgeons were asked to rate only in the context of their respective surgical expertise (i.e., either CRC patients or emergency general surgical patients). A mean relevance score for each concept was calculated using pooled surgeon scores. In addition, stratified mean relevance scores were calculated using only colorectal surgeon scores and emergency abdominal surgeon scores, respectively. We did not ask patients to rate the concepts by relevance, as they were not expected to know the relative importance of health concepts that they had not experienced.

We selected health concepts with an overall or stratified relevance score ≥ 4.0 for inclusion in the conceptual framework. We additionally included health concepts with a relevance score ≥ 3.0 combined with frequency score ≥ 20% or a relevance score ≥ 2.5 combined with frequency score ≥ 50%. As few health concepts did not receive a frequency score due to late entry to the concept pool, we allowed re-entry of moderately relevant concepts (relevance score between 2.5 to 4) if consensus was established between all authors (JC, IGO, TW, VB).

### Statistics

Patient and expert survey data were manually entered into a designated online server (SurveyXact, Ramboll Management Consulting, Aarhus) and analyzed with descriptive statistics using R version 4.1.3.

## Results

### Phase 1: concept pool generation

Thirty-five PROMs measuring recovery or HRQOL after abdominal surgery were identified, of which we were able to retrieve 31 (Table 1). A total of 595 specific items were extracted verbatim for content analysis. Seventy-four eligible patients were identified, of which 49 (15 CRC patients and 34 emergency surgery patients) accepted study participation. Table 2 summarizes baseline characteristics and primary reasons for unplanned readmission. The majority of patients were readmitted within 14 days of discharge (median 5 days, IQR 3–8 days). The median recall time (i.e., time from readmission to interview) was 7.5 months (IQR 4.7–9.6 months). The final concept pool consisted of 58 distinct health concepts, of which six were added by patients or health professionals: ‘Pus from rectum,’ ‘generalized edema,’ ‘weight loss (subjectively assessed),’ ‘wound bleeding,’ ‘stool consistency’ and ‘follow-up from health professionals.’ The majority of identified concepts were categorized as symptoms (*N* = 40), which were subdivided into six sub-domains: ‘Circulatory/respiratory,’ ‘gastrointestinal,’ ‘urogenital,’ ‘general,’ ‘surgical wound,’ and ‘stoma.’ Health concepts related to emotions, cognitive function, and sleep disturbances were categorized as functional impairments, as these were considered to be consequences of upstream symptoms. Online resource 3 summarizes the health concept extraction and categorization process.

**Table 1:** Patient-reported outcome measures (PROMs) evaluated for content (left column)

PROM	Items	Circulatory/ respiratory symptoms	Gastro-intestinal symptoms	Urogenital symptoms	General symptoms	Surgical wound	Stoma	Functional status	General health perception
Well-being index for surgical patients (WISP)[19]	25		x		x			x	
Quality of Recovery-9 (QoR-9)[20]	9	x	x	x	x			x	
Post-discharge Surgical Recovery (PSR)[21]	15				x			x	x
Quality of Recovery-40 (QoR-40)[22]	40	x	x		x			x	
Abdominal Surgery Impact Scale (ASIS)[23]	18		x		x	x		x	
Convalescence and recovery evaluation (CARE)[24]	20		x		x			x	
Recovery Index-10 (RI-10)[25]	10		x		x			x	x
Short-form 36 (SF-36)[25]	36				x			x	x
Postoperative Recovery Profile (PRP)[26]	19		x		x			x	
Postoperative Quality of Life (PQL)[27]	14		x		x			x	x
Functional Recovery Index (FRI)[28]	14				x			x	
Post-General Surgery quality of life (PGSQL)[29]	30	x	x		x			x	x
Surgical Recovery Scale (SRS)[30]	13				x			x	
Postoperative Recovery Index (PoRI)[31]	35		x		x			x	x
Quality of Recovery-15 (QoR-15)[32]	15	x	x		x			x	x
EuroQOL-5D (EQ-5D)[33]	5				x			x	
Cleveland Global Quality of Life (CGQL)[34]	3				x				x
PROMIS 10[35]	10				x			x	x
WHO Disability Assessment Schedule 2.0 (WHODAS 2.0)[36]	15							x	x
Core Outcome Measures Index – hernia (COMA-hernia)[37]	20		x	x	x	x		x	x
Post–liver transplant quality of life (pLTQ)[38]	40		x		x	x		x	x
fecal incontinence quality of life (FIQL)[39]	14		x		x			x	
Carolinas Comfort Scale (CCS)[40]	11	x			x			x	
Esophago-Gastric surgery and Quality of Dietary life (EGQD)[41]	14		x		x			x	
Low Anterior Resection Syndrome (LARS-score)[42]	5		x						
Activities Assessment Scale (AAS)[43]	15							x	
Community Health Activities Model Program for Seniors (CHAMPS)[44]	41							x	
EORCT quality of life of cancer patients core questionnaire (QLQ-C30)[45]	30	x	x		x			x	x
EORCT colorectal module (QLQ-CR29)[45]	33		x	x	x		x	x	x
The colostomy impact score (CI score)[46]	7						x		
Bluebelle wound healing questionnaire (WHQ)[47]	19				x	x			
*Total count*	595	6	18	3	26	4	2	27	14

The table shows number of extracted items per PROM. In addition, the specific health domains covered by each PROM are visualized. Numbers are count

**Table 2:** Baseline characteristics of included patients

	Overall (*N* = *49*)	CRC (*N* = *15*)	Emergency (*N* = *34*)
*N*	49	15	34
Gender, male (%)	23 (46.9)	11 (73)	12 ( 35)
Age, years	68.8 (57.2–74.3)	74.3 (68.3–79.3)	64.9 (53.3–71.9)
*Smoking (%)*			
No	34 (69)	12 (80)	22 (65)
Yes	10 (20)	3 (20)	7 (21)
NA	5 (10)	0 (0)	5 (15)
*Alcohol, units (%)*			
0	21 (43)	6 (40)	15 (44)
0–14	21 (43)	8 (53)	13 (38)
> 14	2 (4)	1 (7)	1 (3)
NA	5 (10)	0 (0)	5 (15)
*ASA (%)*			
1	2 (4.1)	0 (0)	2 (6)
2	27 (55)	6 (40)	21 (62)
3	20 (41)	9 (60)	11 (32)
*Surgery indication (%)*			
Colon cancer	11 (22)	11 (73)	0 (0)
Ileus	21 (43)	0 ( 0)	21 (62)
Intraabdominal abscess	1 (2)	0 ( 0)	1 (3)
mesenteric ischemia	1 (2)	0 ( 0)	1 (3)
Perforated gastrointestinal tract	10 (20)	0 ( 0)	10 (29)
Postoperative bleeding	1 (2)	0 ( 0)	1 ( 3)
*Rectal cancer*	4 (8)	4 (27)	0 (0)
*Procedure performed (%)*			
Diagnostic laparoscopy	6 (12)	0 (0)	6 ( 17.6)
ELAPE	1 (2)	1 (7)	0 ( 0.0)
Explorative laparotomy	28 (57)	0 (0)	28 ( 82.4)
Left colectomy	3 ( 6)	3 (20)	0 ( 0.0)
Low anterior resection	1 ( 2)	1 (7)	0 ( 0.0)
Right colectomy	8 (16)	8 (53)	0 ( 0.0)
TaTME	2 (4)	2 (13)	0 ( 0.0)
Stoma (%)	16 (33)	6 (40)	10 (29)
Length of surgery, minutes	143 (112–209)	180 (141–242)	134 (105–157)
LOS	5 (3–8)	4 (3–6)	5 (4–8)
*Day of readmission*	5.0 (3–11)	5 [3–13]	6 (4–11)
Reason for readmission (%)			
Abdominal pain/unspecific	6 (12)	2 (13)	4 (12)
Bleeding	4 (8)	3 (20)	1 (3)
Dehydration/fluid imbalance	8 (16)	2 (13)	6 (18)
Intestinal obstruction	4 (8)	2 (13)	2 (6)
Obstipation	7 (14)	1 (7)	6 (18)
Other (not related to surgery)	3 (6)	0 (0)	3 (9)
Respiratory insufficiency	1 (2)	0 (0)	1 (3)
Sepsis	3 (6)	3 (20)	0 (0)
Surgical site infection	9 (18)	1 (7)	8 (24)
Venous thromboembolism	2 (4)	0 (0)	2 (6)
Wound rupture	2 (4)	1 (7)	1 (3)
*Clavien-Dindo classification (%)*			
1	7 (14)	4 (27)	3 (9)
2	31 (63)	8 (53)	23 (68)
3	10 (20)	3 (20)	7 (21)
4	1 (2)	0 (0)	1 (2)
Length of readmission, days	1 (0–4)	2 (1–5)	1 (0–3)

Patients are stratified according to surgery type (major emergency abdominal surgery vs colorectal cancer surgery). Numbers are count (%) for categorical variables and median (IQR) for continuous variables

*CRC* Colorectal cancer, Emergency: Major emergency abdominal surgery, *ELAPE* Extralevator abdominoperineal excision, *TaTME* Transanal total mesorectal excision, *LOS* length of stay during index hospitalization (i.e., from index surgery to discharge), day of readmission: Time in days between discharge and first unplanned readmission, *CDC* Clavien-Dindo classification, *NA* Missing value

Gastrointestinal symptoms were reported in 80% of the patients. ‘Fatigue’ was the most frequent single health concept occurring before readmission (69%) followed by ‘Worry from family members’ (67%) and ‘reduced ability to perform activities of daily living’ (60%) (online resource 4 and 5). We did not calculate frequency scores for stoma-related health concepts, as we only included 16 patients readmitted with a stoma.

### Phase 2: health concept selection

**Fig. 2:**
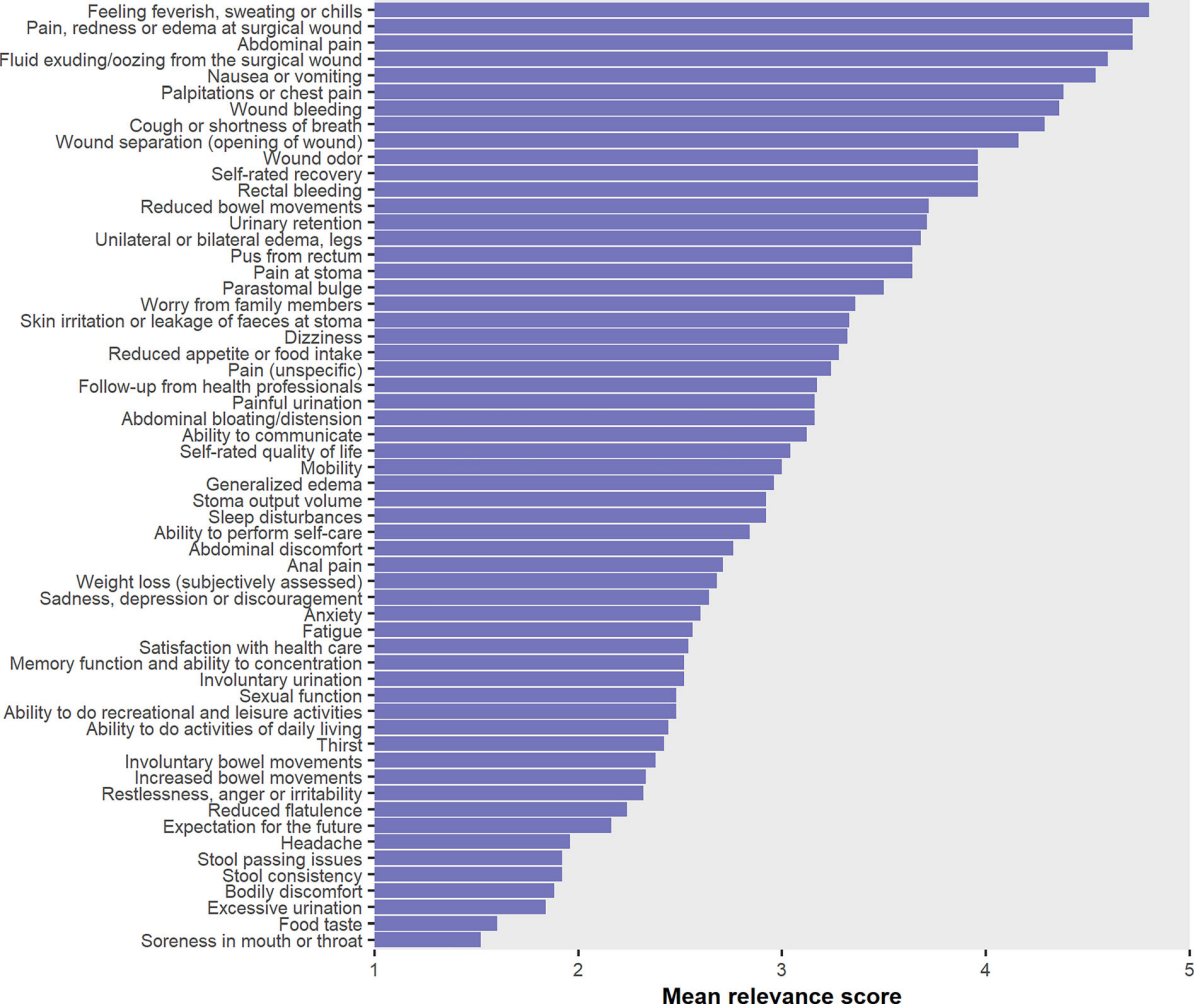
Relevance of health concepts rated by 13 colorectal cancer surgeons and 12 emergency abdominal surgeons in the context of detecting postoperative adverse events after discharge. The relevance score was rated on a 5-point Likert scale (1 = irrelevant, 5 = very relevant)

**Fig. 3:**
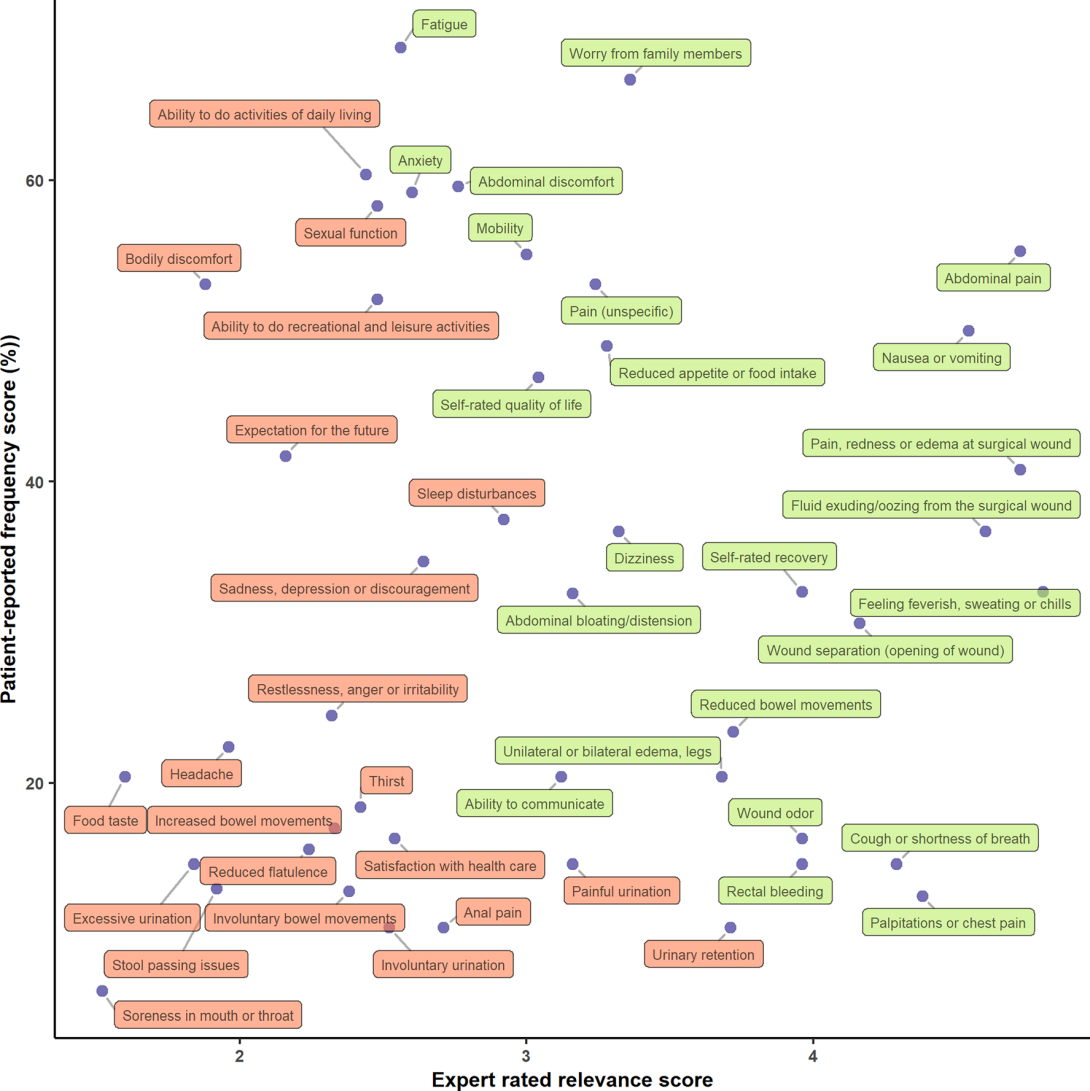
Scatter plot of surgeon-rated relevance score (x-axis) vs patient-rated frequency score (y-axis). The green color corresponds to concepts that fulfilled pre-specified selection criteria for inclusion in the conceptual framework. Concepts without frequency scores (i.e. concepts related to stoma issues and concepts introduced late by patients or experts) are not visualized

**Fig. 4:**
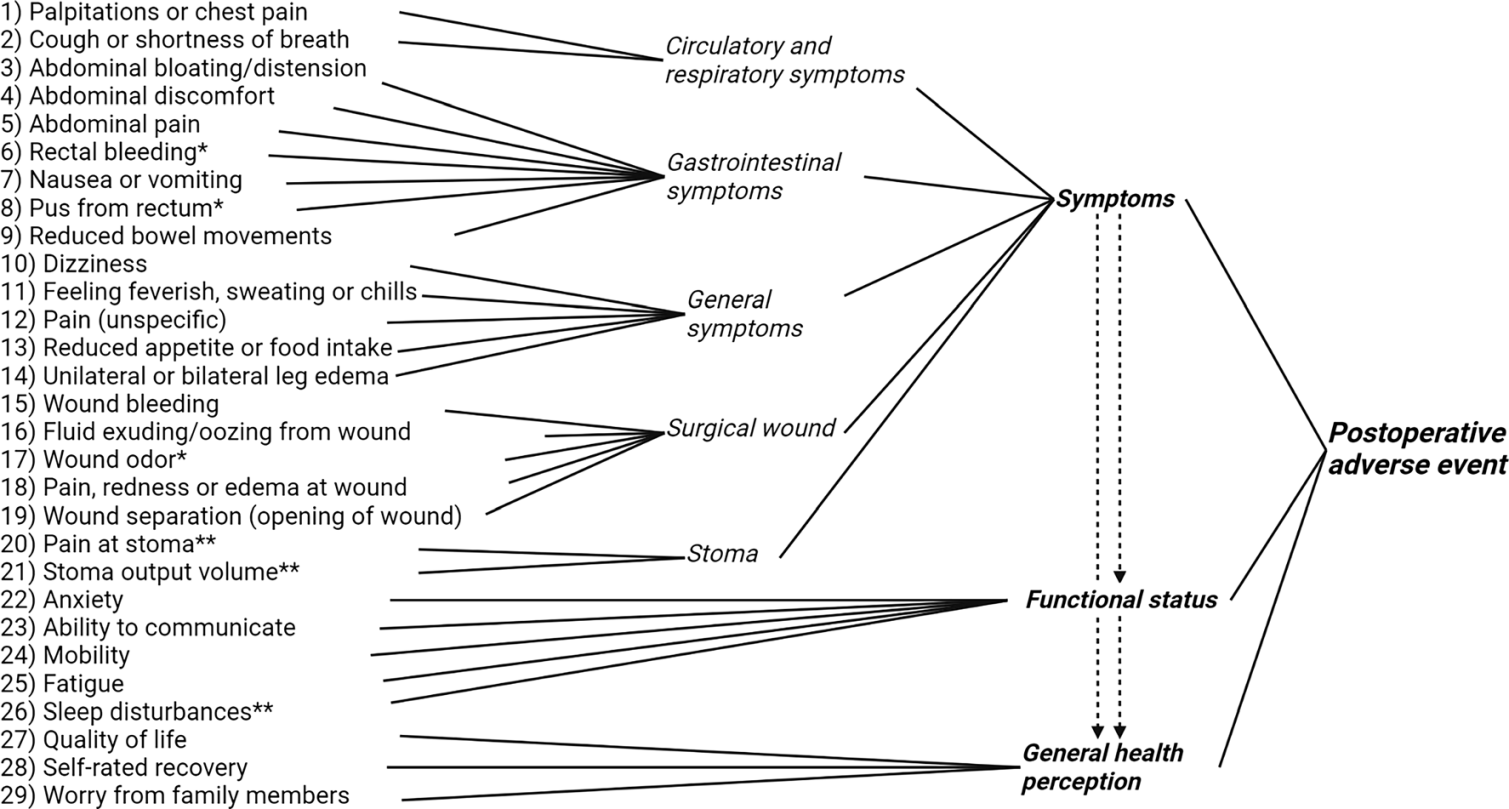
Final conceptual framework of selected health concepts. Individual health concepts (numbered 1–29) are grouped into domains. A causal downstream association was hypothesized between symptoms, functional status, and general health perception (dotted arrows). *only relevant for colorectal cancer patients, **re-entered concepts with moderate relevance (relevance score 2.5–4)

Twenty-five experts (13 colorectal cancer surgeons and 12 emergency general surgeons) completed the health concept rating survey. The participating experts’ surgical experience ranged from six to > 40 years. The mean relevance score ranged from 1.5 to 4.8 across all health concepts. The highest rated health concepts were ‘feeling of having fever, sweating or chills,’ ‘abdominal pain,’ and ‘pain, redness or swelling at surgical wound’ (mean relevance score 4.80, 4.72, and 4.72, respectively) (Fig. 2). Twenty-six concepts were selected according to the defined selection threshold (Fig. 3, online resource 6). Three excluded concepts with moderate relevance were subsequently re-entered after consensus was established in the author group: ‘Sleep disturbances,’ ‘pain at stoma,’ and ‘stoma output volume.’ The resulting conceptual framework is visualized in Fig. 4.

## Discussion

In this study, we have outlined a conceptual framework of patient-reported issues related to adverse events occurring within two weeks of discharge from major emergency abdominal surgery and CRC surgery. Twenty-nine distinct health concepts covering symptoms, functional impairments, and general health perceptions were identified with integrated inputs from existing literature, readmitted patients, and expert health professionals. The resulting framework provides data suitable for ensuring content validity of subsequent operationalizations, e.g., ePROM surveillance systems aimed at early detection of adverse events after discharge from major abdominal surgery.

A characteristic pattern was noted when comparing patient-reported frequency scores with expert-rated relevance scores (Fig. 3). Overall, symptoms related to specific complications (e.g., ‘wound separation’) tended to receive high expert relevance ratings, while non-specific symptoms and functional impairments (e.g,. ‘fatigue’) only received moderate or low relevance scores. In contrast, patients reported broader and non-specific concepts occurring frequently prior to readmission. Specific symptoms may be highly predictive of a few, severe complications, while broader health domains may be sensitive, but not specific, to a diverse range of postoperative adverse events. Hence, a strength of this study is the integration of both frequency and relevance scores in the conceptual framework establishment and content selection process, likely including both sensitive and specific concepts. The integrated relevance and frequency scores may also support the item selection and development of other PROM surveillance systems intended for early detection of specific post-discharge complications. In such cases, researchers should consider items with high relevance ratings to ensure content validity and thus sensitivity and specificity of the instrument.

Consistent with our findings, surgical site infection, intestinal obstruction, bleeding, dehydration/fluid imbalance (including stoma-related high output), pneumonia, and venous thromboembolism have been reported as the most frequent causes of readmission after CRC surgery and emergency abdominal surgery, most often occurring within 10–14 days of discharge [48, 49]. The majority of symptoms included in this framework cover frequent post-discharge complications.

Several ePROM systems for symptom monitoring have previously been developed for oncological patients receiving chemotherapy [50–53]. Basch et al. showed that self-monitoring of 12 common symptoms via the Symptom Tracking and Reporting (STAR) system between scheduled hospital visits improved HRQOL, quality-adjusted survival, and readmission rates compared to usual follow up in patients with advanced solid cancers [52]. Web-mediated follow-up has also been shown to be superior to routine surveillance with computed-tomography scans at detecting cancer relapse and overall survival in patients treated for lung cancer [54]. However, few ePROM monitoring systems have been developed for detecting adverse events in a post-surgical setting. One electronic symptom monitoring system with individually-tailored self-management advice has been developed for patients undergoing surgery for upper gastrointestinal cancer [55]. However, the ePROM was intended for weekly completion, focusing on adverse events occurring within eight weeks of discharge [56]. A higher data sampling rate might be necessary for detecting early complications. Accordingly, minimizing ePROM complexity is likely crucial to reduce patient burden and maintain high response rates over time [13]. This developed framework provides content for further testing of a novel remote ePROM surveillance system with repeated symptom severity ratings and automated alerts to patients and hospital staff if deteriorations are detected.

According to current guidelines, a well-documented conceptual framework is a prerequisite for high-quality evidence of content validity in PROM development [12–14]. Only a few papers have conceptualized postoperative recovery after abdominal surgery, and the majority were based on inputs from a single source or have not been described in detail [12, 24, 57, 58]. Moreover, patients’ perceptions of recovery or HRQOL may differ from their objective health status, although a substantial overlap is plausible. Hence, existing recovery- or HRQOL instruments may not cover all health domains related to surgical complications, possibly resulting in suboptimal prediction of post-discharge complications. This study systematically conceptualized important patient-reported health deteriorations related to post-discharge complications, supporting further operationalization and validation of novel monitoring systems with high predictive power.

The study has some limitations. The target population was defined as patients undergoing either major emergency abdominal surgery or colorectal cancer surgery, and our findings may not be directly transferable to other postoperative trajectories. In addition, differences in postoperative risk profiles may occur between CRC and emergency abdominal surgery, however, this was accounted for by including stratified relevance scores in the selection criteria. Future testing of the final ePROM system may validate the predictive ability in both patient groups. We did not define an a-priori criterion for thematic saturation (i.e., when no further health concepts were added during patient interviews), which is the recommended standard in current guidelines [13]. However, we retrospectively identified all patients readmitted over 12 months at a high-volume surgical center and invited all eligible patients consecutively for study inclusion. Moreover, the patient sample size was larger than the minimum recommendations [13]. The retrospective method of patient identification, however, included a considerable risk of recall bias due to the significant time delay between readmission and interview. Hence, less notable symptoms may not be recalled by some patients due to this time delay. A substantial selection bias was also expected, as we were not able to include perspectives from patients who died after a postoperative complication or patients who were not readmitted due to a post-operative adverse event. Moreover, we did not include a control group (i.e. non-readmitted patients), and it is plausible that frequently occurring symptoms are also expected during a normal recovery trajectory. Additional item selection based on large-scale, prospective testing are required to ensure sufficient specificity of a future ePROM.

In conclusion, this work provides the conceptual framework for a future ePROM measuring health deteriorations related to postoperative complications after major emergency abdominal surgery and CRC surgery. As is intended by our study group, future development steps include operationalization of identified domains into items and scales with subsequent large-scale studies validating the psychometric properties and predictive accuracy.

### Supplementary Information

Below is the link to the electronic supplementary material.

Supplementary file1 (DOCX 166 kb)

Supplementary file2 (DOCX 13 kb)

Supplementary file3 (DOCX 13 kb)

Supplementary file4 (DOCX 85 kb)

Supplementary file5 (DOCX 619 kb)

Supplementary file6 (DOCX 231 kb)

## Abbreviations

CRC: Colorectal cancer
EHR: Electronic health record
ePROM: Electronic patient-reported outcome measure
HRQOL: Health-related quality of life
ICD-10: World Health Association’s international classification of disease, version 10
ICF: World Health Association’s International Classification of Functioning, Disability, and Health
NCSP: Nordic Medico-Statistical Committee classification of Surgical Procedures
OMEGA: Optimizing major emergency abdominal surgery
PROM: Patient-reported outcome measure
