# Supplementary material for: A Content Framework of a Novel Patient-Reported Outcome Measure for Detecting Early Adverse Events After Major Abdominal Surgery
Source: World J Surg. 2023 Aug 23;47(11):2676–87. doi: 10.1007/s00268-023-07143-w (PMC10545596; doi:10.1007/s00268-023-07143-w)

Online Resource 1:

Patient selection citeria and flowchart. Diagnosis codes WHO’s international classification of disease version 10 (ICD-10). Procedure codes are the Danish version of the Nordic Medico- Statistical Committee classification of surgical procedures (NCSP) codes. ZUH: Zealand University Hospital Koege. OMEGA: ‘Optimizing major emergency abdominal surgery’.


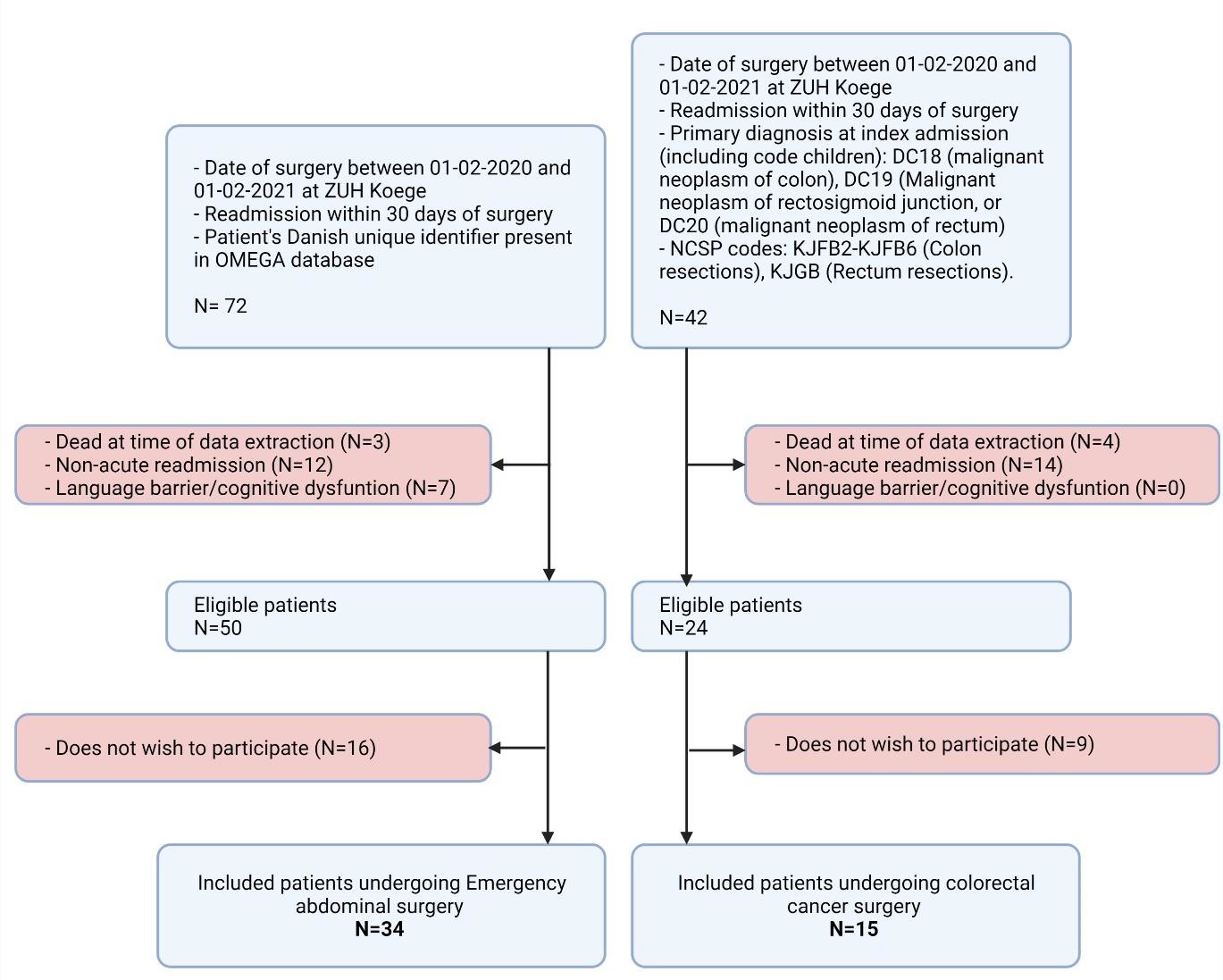

Supplement: Supplementary file 1 — Supplementary file1 (DOCX 166 kb) [file 268_2023_7143_MOESM1_ESM.docx]
