# Supplementary material for: A Content Framework of a Novel Patient-Reported Outcome Measure for Detecting Early Adverse Events After Major Abdominal Surgery
Source: World J Surg. 2023 Aug 23;47(11):2676–87. doi: 10.1007/s00268-023-07143-w (PMC10545596; doi:10.1007/s00268-023-07143-w)
Supplement: Supplementary file 2 — Supplementary file2 (DOCX 13 kb) [file 268_2023_7143_MOESM2_ESM.docx]

Online Resource 2:

Interview guide for semi-structured interviews of included patients. Questions were asked in Danish. Before the interview, the study investigator emphasized that the primary interest was the patient’s perspective on their health in the days prior to readmission.

1. *Please describe, in your own words, why you were readmitted to the hospital?*
2. *How were you feeling in the days and hours before your readmission?*
3. *What made you contact the hospital?*
4. *Which symptoms or functional impairments do you think are relevant to measure to detect deteriorations in patients’ health status at home?*
