# Supplementary material for: A Content Framework of a Novel Patient-Reported Outcome Measure for Detecting Early Adverse Events After Major Abdominal Surgery
Source: World J Surg. 2023 Aug 23;47(11):2676–87. doi: 10.1007/s00268-023-07143-w (PMC10545596; doi:10.1007/s00268-023-07143-w)
Supplement: Supplementary file 3 — Supplementary file3 (DOCX 13 kb) [file 268_2023_7143_MOESM3_ESM.docx]

Online Resource 3:

Summary of the health concept generation phase (phase 1). The left column corresponds to item content from existing patient-reported outcome measures (PROM) enriched with patient and expert inputs (bottom sections). Concepts marked with one star (*) were assessed as unfeasible for self- reporting and not mapped. Concepts marked with two stars (**) were assessed as concepts requiring measurements and excluded. Middle and right column corresponds to the concept pool after the refinement and categorization procedure. Concepts are numbered according to the order presented for relevance rating (phase 2).

| **LITERATURE REVIEW AND EXPERT/PATIENT INPUT** | **MAPPED HEALTH CONCEPT**  **DOMAIN** | **MAPPED HEALTH CONCEPT** |
| --- | --- | --- |
| ****Biological function (NA)** | **-** | **-** |
| **Increased body temperature (Tp>38,0) (NA) | - | - |
| **Daily weight (NA) | - | - |
| **Pain medication intake (NA) | - | - |
| **Circulatory and respiratory symptoms (ICD R00-R09)** |  |  |
| Palpitations (R00.2) | **Circulatory and respiratory symptoms** | 1) Palpitations or chest pain |
| Cough (R05) | **Circulatory and respiratory symptoms** | 2) Cough or shortness of breath |
| Breathing (R06) | **Circulatory and respiratory symptoms** | 2) Cough or shortness of breath |
| Chest pain (R07) | **Circulatory and respiratory symptoms** | 1) Palpitations or chest pain |
| **Gastrointestinal symptoms (ICD R1 + K)** |  |  |
| Abdominal pain (R100) | **Gastrointestinal symptoms** | 3) Abdominal pain |
| Rectal/anal/perineal pain (R10.2) | **Gastrointestinal symptoms** | 4) Anal pain |
| Abdominal discomfort (R104) | **Gastrointestinal symptoms** | 5) Abdominal discomfort |
| Nausea (R119B) | **Gastrointestinal symptoms** | 6) Nausea or vomiting |
| Vomiting (R119C) | **Gastrointestinal symptoms** | 6) Nausea or vomiting |
| Abdominal distension or bloating (R14) | **Gastrointestinal symptoms** | 7) Abdominal bloating/distension |
| Flatulence (R149) | **Gastrointestinal symptoms** | 8) Reduced flatulence |
| Faecal incontinence (R15) | **Gastrointestinal symptoms** | 9) Involuntary bowel movements |
| Frequency of bowel movements (R194) | **Gastrointestinal symptoms** | 10) Reduced bowel movements, 11) Increased bowel  movements |
| *Mucus in stool (R19.5) | - | - |
| Defecation (R19.8) | **Gastrointestinal symptoms** | 13) Stool passing issues |
| Rectal tenesmus (incomplete defecation) (R198E) | **Gastrointestinal symptoms** | 13) Stool passing issues |
| Gastrointestinal hemorrhage (K92.2) | **Gastrointestinal symptoms** | 14) Rectal bleeding |
| Soreness in mouth/throat (NA) | **Gastrointestinal symptoms** | 16) Soreness in mouth or throat |
| **Symptoms and signs involving the nervous and**  **musculoskeletal systems (ICD R25-R29)** | **-** | **-** |
| *Physical coordination (R27) | - | - |
| **Urogenital symptoms (ICD R30-R39**) | **Urogenital symptoms** |  |
| Dysuri (R30) | **Urogenital symptoms** | 17) Painful urination |
| Polyuri (R35) | **Urogenital symptoms** | 18) Excessive urination |
| Urinary incontinence (R32) | **Urogenital symptoms** | 19) Involuntary urination |
| Urinary retention (R33) | **Urogenital symptoms** | 20) Urinary retention |
| **Cognition, perception and emotional state (ICD R4 + G0-G9)** |  |  |
| *Somnolence (R40.0) | - | - |
| Cognitive Function (R41) | **Functional status** | 47) Memory function and ability to concentration |
| Dizzyness (R42) | **General symptoms** | 21) Dizziness |
| Food taste (R43) | **General symptoms** | 22) Food taste |
| *Speech (R47) | - | - |
| Sleep disturbances (G47) | **Functional status** | 52) Sleep disturbances |
| Anxiety (R452B) | **Functional status** | 48) Anxiety |
| Restlessness (R451A) | **Functional status** | 49) Restlessness, anger or irritability |
| Unhappiness (R452A) | **Functional status** | 50) Sadness, depression or discouragement |
| Anger or irritability (R454) | **Functional status** | 49) Restlessness, anger or irritability |
| Nervousness (R450) | **Functional status** | 48) Anxiety |
| *Other emotional states (R458) | - | - |
| **General symptoms (ICD R50-R69)** |  |  |
| Fever (subjective) (R50) | **General symptoms** | 23) Feeling feverish, sweating or chills |
| Headache (R51) | **General symptoms** | 24) Headache |
| Pain, unspecified (R52) | **General symptoms** | 25) Pain (unspecific) |

| Fatigue (R53) | **Functional status** | 51) Fatigue |
| --- | --- | --- |
| Periferal oedema (R60) | **General symptoms** | 26) Unilateral or bilateral edema, legs |
| Appetite (R630) | **General symptoms** | 28) Reduced appetite or food intake |
| Reduced food intake (R633) | **General symptoms** | 28) Reduced appetite or food intake |
| Thirst (R63.8) | **General symptoms** | 29) Thirst |
| General bodily discomfort (R68) | **General symptoms** | 30) Bodily discomfort |
| Chills (R688) | **General symptoms** | 23) Feeling feverish, sweating or chills |
| **Surgical wound (NA)** |  |  |
| *Local skin sensory disturbances (NA) | - | - |
| Incisional pain (NA) | **Surgical wound** | 32) Pain, redness or swelling at surgical wound |
| Local redness (NA) | **Surgical wound** | 32) Pain, redness or swelling at surgical wound |
| Localized swelling (NA) | **Surgical wound** | 32) Pain, redness or swelling at surgical wound |
| *Skin temperature around wound (NA) | - | - |
| Fluid leakage (serous, haemoserous or purulent) (NA) | **Surgical wound** | 33) Fluid exuding/oozing from the surgical wound |
| Wound seperation (NA) | **Surgical wound** | 35) Wound separation (opening of wound) |
| Wound odor (NA) | **Surgical wound** | 36) Wound odor |
| **Stoma (NA)** |  |  |
| *Embarrassing smells from stoma bag (NA) | - | - |
| Leakage of faeces (NA) | **Stoma** | 37) Skin irritation or leakage of faeces at stoma |
| *Consistency of faeces in stoma bag (NA) | - | - |
| Pain related to stoma (NA) | **Stoma** | 38) Pain at stoma |
| Skin problems in relation to the stoma (NA) | **Stoma** | 37) Skin irritation or leakage of faeces at stoma |
| Bulge in relation to the stoma (NA) | **Stoma** | 39) Parastomal bulge |
| Frequency of stoma bag-changes (NA) | **Stoma** | 40) Stoma output volume |
| *Mucocutaneus separation of stoma (NA) | - | - |
| **Functional status (ICF)** |  |  |
| Daily life activities (d23) | **Functional status** | 41) Ability to do activities of daily living |
| Communicating (D3) | **Functional status** | 42) Ability to communicate |
| Mobility, unspecified (d4) | **Functional status** | 43) Mobility |
| Changing and maintaining body position (D410-D429) | **Functional status** | 43) Mobility |
| Carrying, moving and handling objects (d430-d449) | **Functional status** | 43) Mobility |
| Walking and moving (d450-d469) | **Functional status** | 43) Mobility |
| Moving around using transportation (d470-d489) | **Functional status** | 43) Mobility |
| Self-care (d5) | **Functional status** | 44) Ability to self-care |
| Sexual function/activity (b64) | **Functional status** | 45) Sexual function |
| Recreation and leisure (d92) | **Functional status** | 46) Ability to do recreational and leisure activities |
| **General health perception (NA)** |  |  |
| General recovery (NA) | **General health perception** | 53) Self-rated recovery |
| Quality of life (NA) | **General health perception** | 54) Self-rated quality of life |
| Satisfaction with health care (NA) | **General health perception** | 55) Satisfaction with health care |
| Family members worry (NA) | **General health perception** | 56) Worry from family members |
| Patient expectation for future (NA) | **General health perception** | 57) Expectation for the future |
| **CONCEPT ADDITIONS (PATIENTS)** |  |  |
| Shortness of breath (dyspnea) (R06) | **Circulatory and respiratory symptoms** | 2) Cough or shortness of breath |
| Inadequate follow-up from healthcare professionals (NA) | **General health perception** | 58) Follow-up and help from health professionals |
| Excessive sweating (NA) | **General symptoms** | 23) Feeling feverish, sweating or chills |
| Bleeding from wound (NA) | **Surgical wound** | 34) Wound bleeding |
| Change in stool consistency (R19.8) | **Gastrointestinal symptoms** | 12) Stool consistency |
| Discouraged regarding general health (NA) | **Functional status** | 50) Sadness, depression or discouragement |
| **CONCEPT ADDITIONS (EXPERTS)** |  |  |
| Weightloss (subjectively assessed) | **General health perception** | 31) Weight loss (subjectively assessed) |
| Generalized edema (R60.1) | **General health perception** | 27) Generalized edema |
| Pus from rectum(19.5) | **Gastrointestinal symptoms** | 15) Pus from rectum |
| No stool output from stoma (NA) | **Gastrointestinal symptoms** | 10) Reduced bowel movements |
| Exuding wound (NA) | **Surgical wound** | 33) Fluid exuding/oozing from the surgical wound |
| **Increase in pain medication dose (NA) | **-** | **-** |
| Feeling of not being well-informed (NA | **General health perception** | 58) Follow-up and help from health professionals |
