# Supplementary material for: A Content Framework of a Novel Patient-Reported Outcome Measure for Detecting Early Adverse Events After Major Abdominal Surgery
Source: World J Surg. 2023 Aug 23;47(11):2676–87. doi: 10.1007/s00268-023-07143-w (PMC10545596; doi:10.1007/s00268-023-07143-w)

Online Resource 4:

Patient frequency scores. Patient frequency scores are calculated as the proportion of patients who stated that they experienced a deterioration in the respective health concept.


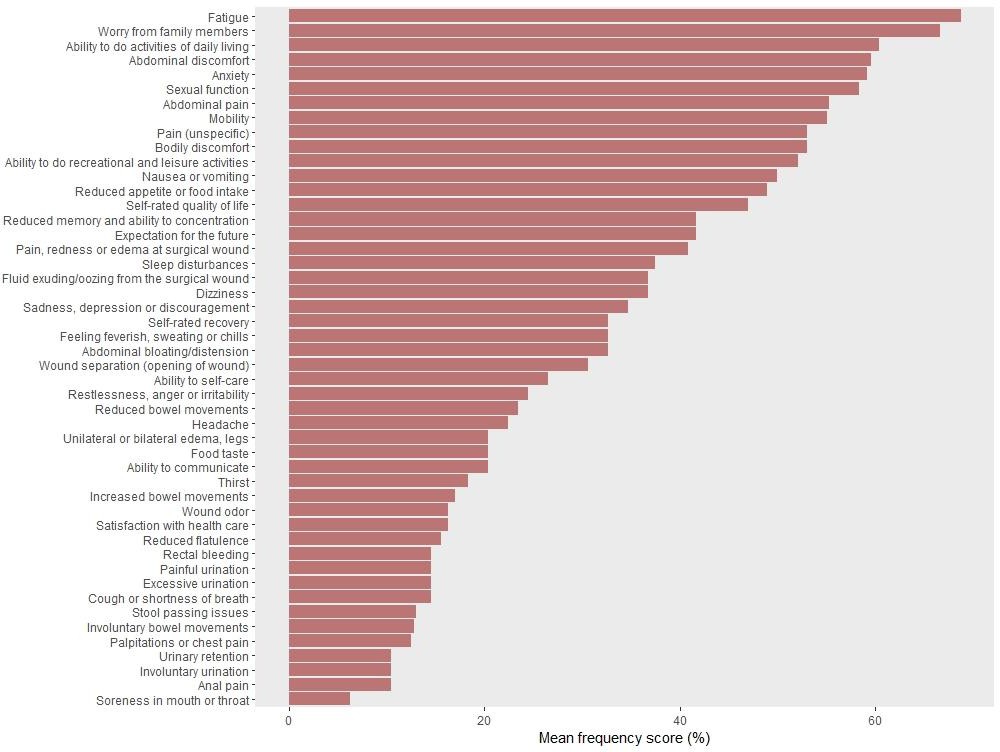

Supplement: Supplementary file 4 — Supplementary file4 (DOCX 85 kb) [file 268_2023_7143_MOESM4_ESM.docx]
