# Supplementary material for: A Content Framework of a Novel Patient-Reported Outcome Measure for Detecting Early Adverse Events After Major Abdominal Surgery
Source: World J Surg. 2023 Aug 23;47(11):2676–87. doi: 10.1007/s00268-023-07143-w (PMC10545596; doi:10.1007/s00268-023-07143-w)

Figure A:

Stratified patient frequency scores. Patient frequency scores are calculated as the proportion of patients who stated that they experienced a deterioration in the respective health concept. Red and blue color corresponds to patients, who underwent major emergency abdominal surgery and colorectal cancer surgery, respectively.

Figure B:

Stratified expert relevance scores. Relevance of health concepts rated by 13 colorectal cancer surgeons (blue) and 12 emergency abdominal surgeons (red) in the context of detecting postoperative adverse events after discharge. The relevance score was rated on a 5-point Likert scale (1= irrelevant, 5=very relevant).


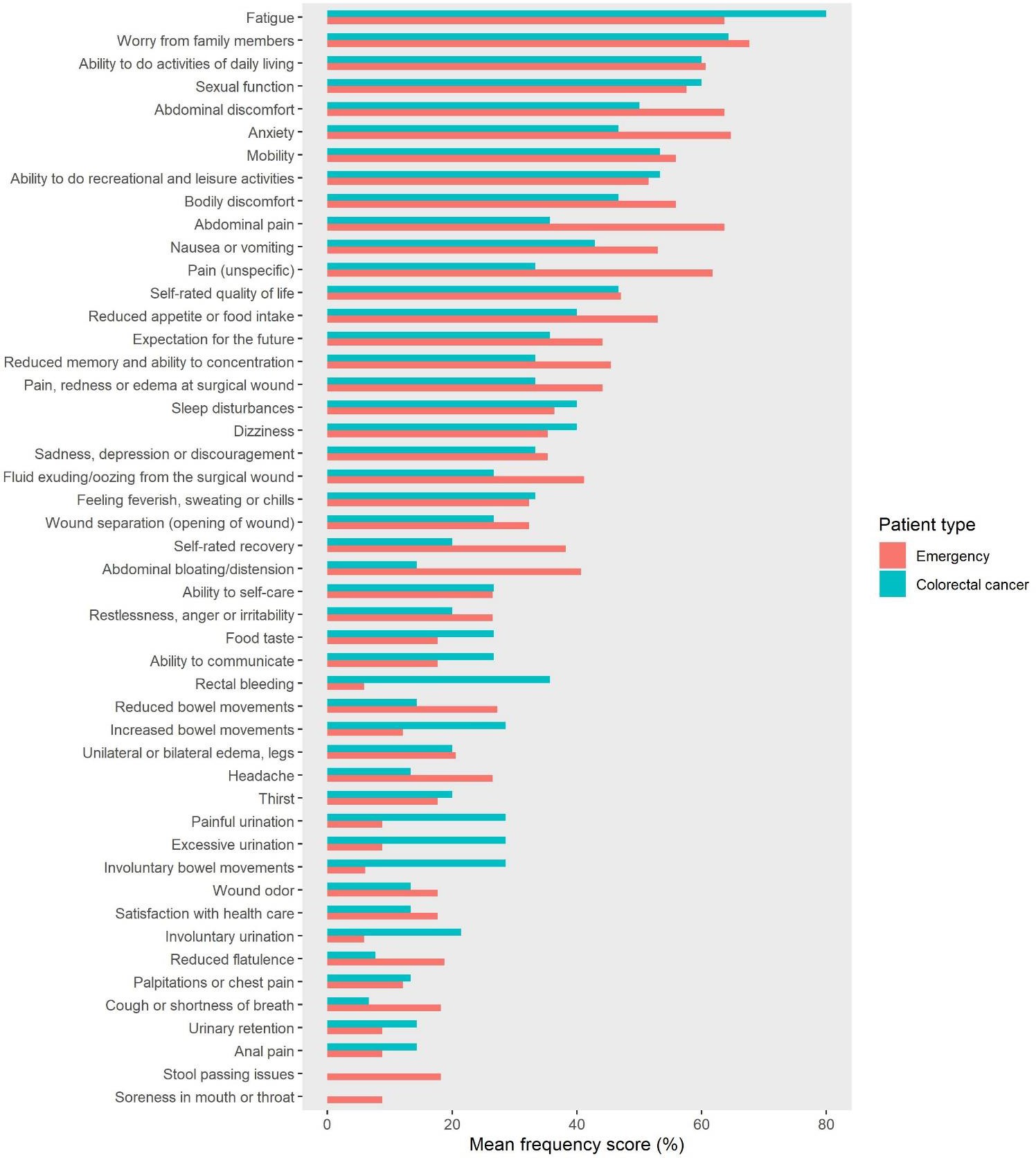


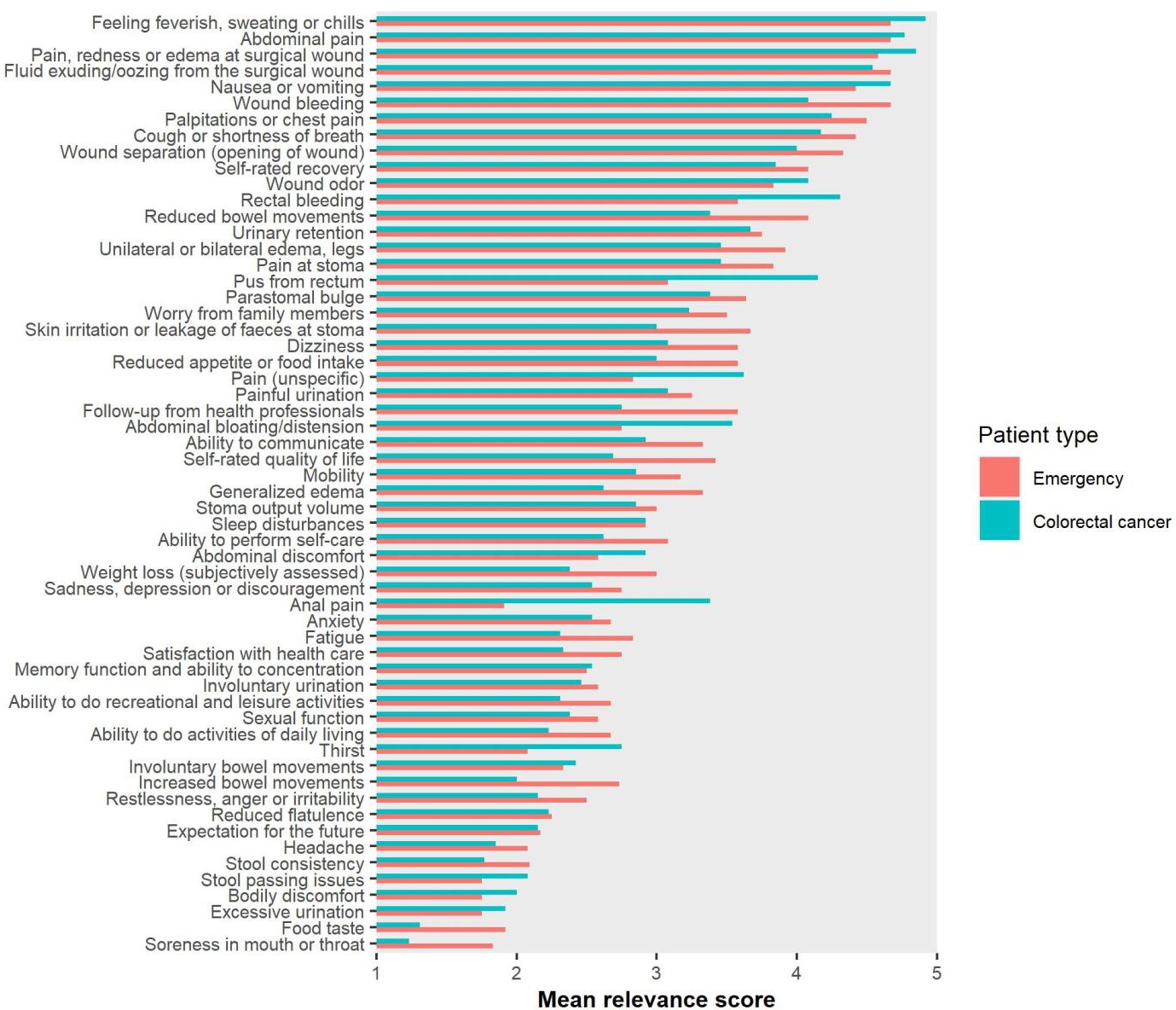

Supplement: Supplementary file 5 — Supplementary file5 (DOCX 619 kb) [file 268_2023_7143_MOESM5_ESM.docx]
