# Supplementary material for: A Content Framework of a Novel Patient-Reported Outcome Measure for Detecting Early Adverse Events After Major Abdominal Surgery
Source: World J Surg. 2023 Aug 23;47(11):2676–87. doi: 10.1007/s00268-023-07143-w (PMC10545596; doi:10.1007/s00268-023-07143-w)

Figure A:

Scatter plot of surgeon-rated relevance score (x-axis) vs patient-rated frequency score (y-axis). Health concepts are colorized according to their corresponding health domain. Concepts without frequency scores (i.e. concepts related to stoma issues and concepts introduced late by patients or experts) are not visualized.

Figure B:

Scatter plot of surgeon-rated relevance score (x-axis) vs patient-rated frequency score (y-axis). Mean relevance and frequency scores of concepts in each health domain are visualized. Concepts without frequency scores (i.e. concepts related to stoma issues and concepts introduced late by patients or experts) are not included in the calculation of mean scores.

Worry from femily members ]

**60**

[ Bodily discamfort I

•

Abilit ta do-activilies of daily living

•


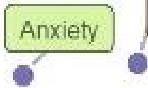


Abdominal d1seomfort

•

Sexua I function

•

Abilit)' lo do recrealional and leisure attivmes


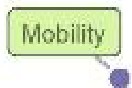


[ Pain (unspecific) ]

•'

[ Reduced appelita or too,d ntake j

•

•

Abdominal pain

.--Na-us-ea -or -vom-1t-mg

**0u**

">,'

**u**

**C**

**GJ 40**

**:I**

**D"**

•

Expectaban fur the future

•

Sett ratad quaht)• of ·,to

Sleep dismroances

•

Pain, rgdn9.ss or ed9ma at surgical wound .

Fluid exudinglQOzing from the surgical wound

**Health concept domain**

a **Circulatory and respiratory symptoms Functional status**

**Gastrointestinal symptoms**

....

-

**1l**

.**G**..**J**

**0**

**CL**

-...

**QI**

**C**

- �

Sadniass, d p ression or d isco.u rag-ami;int

- [ Dizziness ]

[ **AbClomlnaJ blaallng/dlstenslon** ]

S01f-ral9d rgcavgry

'•

I

'•

[ Fooling tevens.h, swaat1ng or ch1l1So ]

'•

**General health perception General symptoms Surgical wound Urogenital symptoms**

:.**^G^**:**^J^**;

**c**I**.**l**.**l

Reslless eVi, nger **or** im1. ilily

Wound separation (opening of wound)]

[ Food taste ]

[ Headache **J**•

- Reduced bowel movements

Unilateral or bilateral edema. legs

**20 'e**

,I Thirst]

Increased bowel movements •

• •

Ab,Iity **lo** communicate

Excess.1ve Lmnatior1 • •

[ Wound odor ]

•

( Cou,gl, or shortne55 of breath ]

- - Reduced flalulenca Sat1sfaction with health care • *.7*

**StiaDl:pl6HIGi&&ia** Involuntary bowel movements

---- ~·-· '--

lnvolunlat,' urination Anal pain

PainfuI urination rRectal bleeding ] •

.,• Palp1tabons or chest pain

[ Unnar,•retention ]


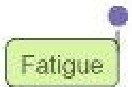


- Soreness m mouth or throat

2 3 4

Expert rated relevarice score


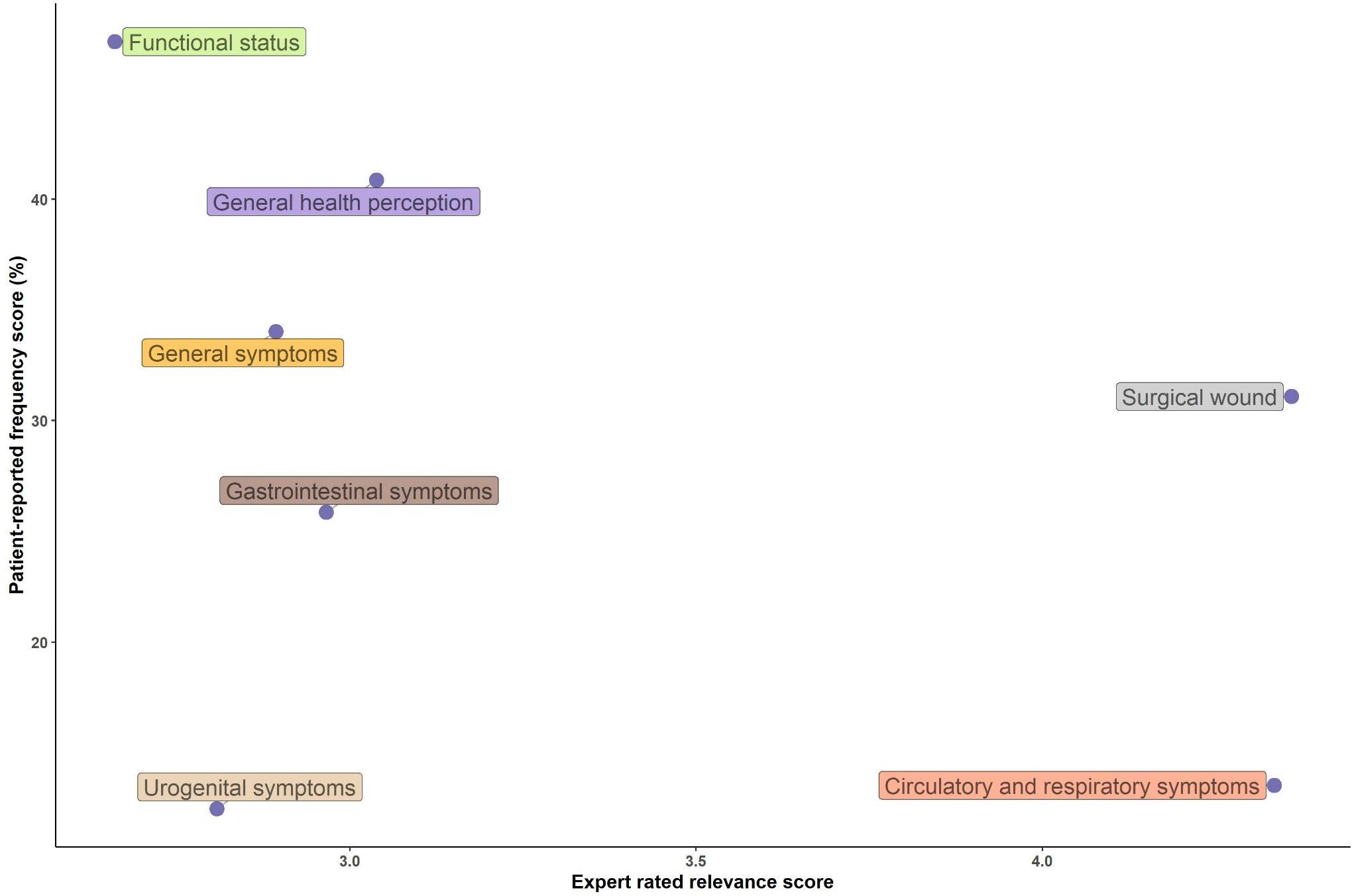

Supplement: Supplementary file 6 — Supplementary file6 (DOCX 231 kb) [file 268_2023_7143_MOESM6_ESM.docx]
